# The individual contributions of *bla*_B_, *bla*_GOB_ and *bla*_CME_ on MICs of β-lactams in *Elizabethkingia anophelis*

**DOI:** 10.1093/jac/dkae137

**Published:** 2024-05-13

**Authors:** Pei-Jing Chen, Mei-Chen Tan, Wei-Cheng Huang, Shu-Yuan Hsu, Te-Li Chen, Chiou-Ying Yang, Shu-Chen Kuo

**Affiliations:** National Institute of Infectious Diseases and Vaccinology, National Health Research Institutes, Miaoli County, Taiwan; Institute of Molecular Biology, National Chung Hsing University, Taichung, Taiwan; National Institute of Infectious Diseases and Vaccinology, National Health Research Institutes, Miaoli County, Taiwan; National Institute of Infectious Diseases and Vaccinology, National Health Research Institutes, Miaoli County, Taiwan; Institute of Population Health Sciences, National Health Research Institutes, Miaoli County, Taiwan; Graduate Institute of Life Sciences, National Defense Medical Center, Taipei, Taiwan; Institute of Molecular Biology, National Chung Hsing University, Taichung, Taiwan; National Institute of Infectious Diseases and Vaccinology, National Health Research Institutes, Miaoli County, Taiwan

## Abstract

**Background:**

The *bla*_B_, *bla*_GOB_ and *bla*_CME_ genes are thought to confer β-lactam resistance to *Elizabethkingia anophelis*, based on experiments conducted primarily on *Escherichia coli*.

**Objectives:**

To determine the individual contributions of β-lactamase genes to increased MICs in *E. anophelis* and to assess their impact on the *in vivo* efficacy of carbapenem therapy.

**Methods:**

Scarless gene deletion of one or more β-lactamase gene(s) was performed in three clinical *E. anophelis* isolates. MICs were determined by broth microdilution. Hydrolytic activity and expressions of β-lactamase genes were measured by an enzymatic assay and quantitative RT–PCR, respectively. *In vivo* efficacy was determined using *Galleria mellonella* and murine thigh infection models.

**Results:**

The presence of *bla*_B_ resulted in >16-fold increases, while *bla*_GOB_ caused 4–16-fold increases of carbapenem MICs. Hydrolysis of carbapenems was highest in lysates of *bla*_B_-positive strains, possibly due to the constitutionally higher expression of *bla*_B_. Imipenem was ineffective against *bla*_B_-positive isolates *in vivo* in terms of improvement of the survival of wax moth larvae and reduction of murine bacterial load. The deletion of *bla*_B_ restored the efficacy of imipenem. The *bla*_B_ gene was also responsible for a >4-fold increase of ampicillin/sulbactam and piperacillin/tazobactam MICs. The presence of *bla*_CME_, but not *bla*_B_ or *bla*_GOB_, increased the MICs of ceftazidime and cefepime by 8–16- and 4–8-fold, respectively.

**Conclusions:**

The constitutionally and highly expressed *bla*_B_ gene in *E. anophelis* was responsible for increased MICs of carbapenems and led to their poor *in vivo* efficacy. *bla*_CME_ increased the MICs of ceftazidime and cefepime.

## Introduction

Nosocomial outbreaks of *Elizabethkingia anophelis* infections have been increasingly reported worldwide. *E. anophelis* isolates are highly resistant to multiple commonly used antibiotics, including β-lactams.^1^ β-Lactam resistance in *E. anophelis* is assumed to result from two intrinsic MBLs (*bla*_GOB_ and *bla*_B_) and one ESBL (*bla*_CME_). Most studies measured the contribution of these genes to β-lactam resistance in *Escherichia coli* (Table S1, available as Supplementary data at *JAC* Online), but not in *E. anophelis*. In this study, deletions of individual β-lactamase genes were performed in *E. anophelis* to determine their contributions to *in vitro* resistance to commonly used β-lactams and to assess their impact on *in vivo* efficacy of carbapenem therapy.

## Methods

### Bacterial isolates for scarless gene deletion

Three isolates (2018C07-210, 2002N07-090 and 2008N05-106) of different clonality from a nationwide surveillance programme (Taiwan Surveillance of Antimicrobial Resistance) were selected. 2018C07-210 and 2002N07-090 both harboured *bla*_B-1_, *bla*_GOB-20_ and *bla*_CME-3_, while 2008N05-106 harboured *bla*_B-3_, *bla*_GOB-3_ and *bla*_CME-3_. Scarless gene deletion of *bla*_GOB_, *bla*_B_ and/or *bla*_CME_ was performed as described in our previous report.^2^ The suicide vector pUT-ermF, with an *erm*(F) gene encoding erythromycin resistance and a *sacB* gene for sucrose counter-selection was used for scarless deletion. The pUT-ermF containing the upstream and downstream regions of the target gene was amplified in *E. coli* S17–λpir, which was then conjugated with *E. anophelis*. Transconjugants were screened first in LB agar with 250 mg/L erythromycin and then in LB agar with 10% sucrose and without NaCl. Seven mutants were obtained from each parent strain (Table 1). Mutant and parent strains were subjected to broth microdilutions^3^ of various β-lactams and levofloxacin (as a control) to determine changes of MICs.

**Table 1.** MICs of different antibiotics in three clinical strains of *E. anophelis* and their gene-edited mutants

Isolates	Presence of intrinsic β-lactamase gene			MIC (mg/L) of 2018C07-210, 2002N07-090 and 2008N05-106							
	*bla*_B_	*bla*_GOB_	*bla*_CME_	IPM	MEM	CAZ	FEP	SAM	TZP	ATM	LEV
WT	+	+	+	64/64/32	>64/>64/64	256/256/256	32/32/32	128/128/64	8/8/8	>128/>128/>128	64/1/64
Δ*bla*_B_		+	+	8/16/8	16/32/16	256/256/256	32/32/32	4/8/4	0.5/0.5/0.5	>128/>128/>128	64/1/64
Δ*bla*_GOB_	+		+	64/64/32	>64/>64/64	256/256/256	32/32/32	128/128/64	8/8/8	>128/>128/>128	64/1/64
Δ*bla*_CME_	+	+		64/64/64	>64/>64/64	32/32/32	4/8/4	128/128/64	8/4/8	>128/>128/>128	64/1/64
Δ*bla*_B_ Δ*bla*_GOB_			+	1/1/0.5	4/4/2	256/256/256	32/32/32	4/4/2	0.5/0.5/0.5	>128/>128/>128	64/1/64
Δ*bla*_B_ Δ*bla*_CME_		+		8/8/8	16/16/16	32/32/16	4/8/4	4/4/2	0.5/0.5/0.25	>128/>128/>128	64/1/64
Δ*bla*_GOB_ Δ*bla*_CME_	+			64/32/32	>64/>64/64	32/32/16	4/8/4	128/128/64	8/4/4	>128/>128/>128	64/1/64
Δ*bla*_B_Δ*bla*_GOB_ Δ*bla*_CME_				1/1/0.5	4/4/2	16/32/16	4/8/8	4/4/2	0.25/0.25/0.5	>128/>128/>128	64/1/64

IPM, imipenem; MEM, meropenem; CAZ, ceftazidime; FEP, cefepime; SAM, ampicillin/sulbactam; TZP, piperacillin/tazobactam; ATM, aztreonam; LEV, levofloxacin.

### β-Lactamase activity

Crude extracts of bacteria at stationary phase were prepared by disruption of bacteria by sonification (2 min at 2 s of sonication and 4 s of rest at 30% amplitude) with a Vibra-Cell VCX 600 (Sonics & Materials, Inc.) and centrifugation for 10 min at 15 000 **g** at 4°C.^4^ The crude extracts of different strains with the same protein concentration were mixed with β-lactams in phosphate buffer (0.1 M, pH 7) with 50 mM ZnSO_4_ at room temperature. The total protein concentration was measured by a BCA protein assay. The hydrolysis of antibiotics was monitored by SpectraMax^®^ M2 (Molecular Devices) for 10 min. The molar extinction coefficients (Δɛ) were as follows: Δɛ_297_ = 8621 M^−1^ cm^−1^ for imipenem and Δɛ_297_ = 5900 M^−1^ cm^−1^ for meropenem.

### Real-time quantitative RT–PCR (qRT–PCR)

Expression levels of β-lactamase genes and 16S rRNA in *E. anophelis* strains were measured by qRT–PCR. Bacteria at mid-log phase were incubated in LB broth with or without imipenem (½ of MIC). The purification of RNA, removal of genomic DNA, and conversion to cDNA were conducted as described in a previous report.^5^ Samples were subjected to qRT–PCR with the reaction mixture containing Luminaris Color HiGreen qPCR Master Mix (Thermo Scientific). Cycling parameters were as follows: 1 cycle of 95°C for 10 min; 40 cycles of 95°C for 15 s, 60°C for 1 min; and 1 cycle of 95°C for 15 s, 60°C for 1 min, and 95°C for 15 s. Expression level results were normalized to the transcription levels of 16S rRNA. The relative expression level was calculated by dividing the expression level of the target β-lactamase gene by that of *bla*_GOB_ in the WT strain without the addition of imipenem.

### Galleria mellonella model

*G. mellonella* was reared at the National Health Research Institutes of Taiwan. The experiment was conducted as described previously.^6–8^ Briefly, a 10 μL aliquot of *E. anophelis* containing an inoculum of 10^4^ cfu was injected into the haemocoel of each caterpillar via the most caudal left proleg. Imipenem (12.5 mg/kg) was given in 10 μL injections into another proleg. After injection, the caterpillars were incubated in plastic containers at 37°C. The number of dead caterpillars was recorded twice daily for 72 h.

### Thigh infection model

Seven-week-old C57BL/6J mice were rendered neutropenic with cyclophosphamide before inoculation, following a previously described protocol.^9^ A 30 μL aliquot containing *E. anophelis* (10^7^ cfu) was injected into each of the two rear thighs of each mouse. Imipenem (100 mg/kg/day) was administered 2 and 14 h after inoculation. The mice were sacrificed 24 h after inoculation. Bacterial counts were determined by plating the homogenized tissue in serial 10-fold dilutions on brain heart infusion agar.

## Results and discussion

Table 1 illustrates that compared with isolates without any known β-lactamase gene (Δ*bla*_B_Δ*bla*_GOB_Δ*bla*_CME_), those carrying *bla*_B_ exhibited >16-fold increases of imipenem and meropenem MICs; *bla*_GOB_ resulted in only 8–16- and 4–8-fold increases, respectively. The *bla*_B_ gene was also responsible for 32- and 8–32-fold increases of ampicillin/sulbactam and piperacillin/tazobactam MICs, respectively. However, *bla*_GOB_ had no effect on the MICs of these two antibiotics. The presence of *bla*_CME_, but not *bla*_B_ or *bla*_GOB_, increased the MICs of ceftazidime and cefepime by 8–16- and 4–8-fold, respectively. These WT isolates exhibited similar MIC values of different β-lactams and similar correlations between the presence of β-lactamase genes and increased MICs. The MICs of aztreonam and ceftazidime in isolates without *bla*_CME_, *bla*_B_ and *bla*_GOB_ remained high, indicating the presence of other mechanisms, i.e. low outer membrane permeability, reduced affinity of PBPs, unknown β-lactamases or overexpression of efflux pumps.

**Figure 1.**
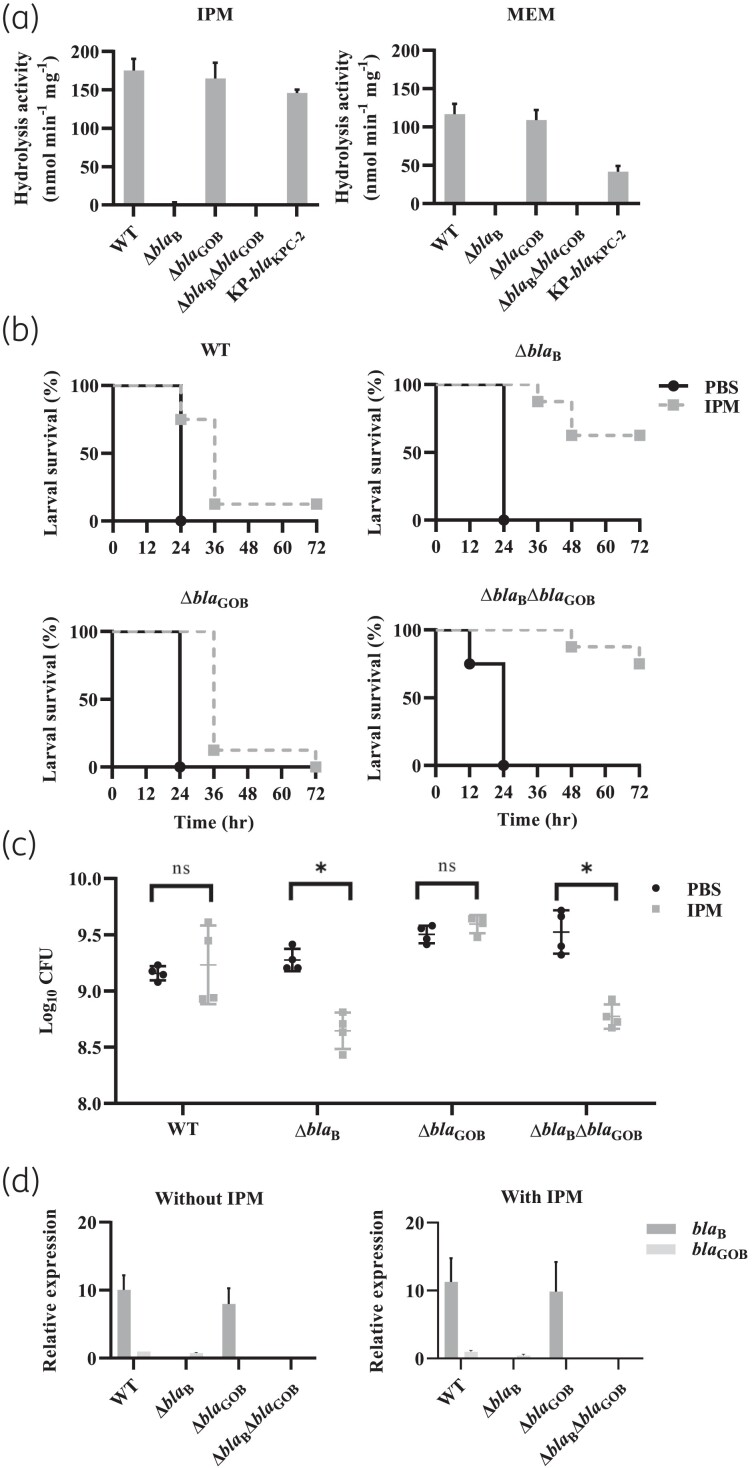
Hydrolytic activity on carbapenems, *in vivo* efficacy of imipenem monotherapy, and relative expression levels of β-lactamase genes in clinical *E. anophelis* isolates and their gene-edited mutants. (a) Hydrolytic activity against imipenem and meropenem in 2018C07-210, its gene-edited mutants, and *Klebsiella pneumoniae* harbouring *bla*_KPC_ (KP-*bla*_KPC_). The *y*-axis indicates the amount of the carbapenem hydrolysed (nmol) per min and per mg of total protein. (b) Survival of *G. mellonella* given imipenem monotherapy using 2008N05-106 and its gene-edited mutants. (c) Bacterial loads in mice given imipenem monotherapy in a thigh infection model using 2008N05-106 and its gene-edited mutants. (d) Relative expression levels of β-lactamase genes in 2018C07-210 and its gene-edited mutants determined by qRT–PCR. The relative expression level was calculated by dividing the expression level of the target β-lactamase gene by that of *bla*_GOB_ in the WT strain without addition of imipenem. IPM, imipenem; MEM, meropenem; ns, not significant; *P < 0.05.

An enzymatic assay (Figure 1a) showed that the 10 min hydrolysis rates of imipenem and meropenem were highest in lysates of *bla*_B_-positive strains. The deletion of *bla*_B_ almost eliminated hydrolytic activity. In contrast, *bla*_GOB_ was only associated with limited hydrolysis of carbapenems. Imipenem did not prolong the survival of wax moth larvae infected with *bla*_B_-positive isolates, including WT or *bla*_GOB_-deleted strains. Furthermore, imipenem was effective among larvae infected with a *bla*_B_-deleted mutant (Figure 1b). A similar result was observed in the murine thigh infection model (Figure 1c). The reduction of bacterial load by imipenem was statistically significant in murine thighs infected by the *bla*_B_-deleted mutant, but not by *bla*_B_-positive isolates.

We hypothesized that the different resistance phenotypes of *bla*_B_- and *bla*_GOB_-positive isolates may result from dissimilar expression levels, and therefore determined their expression levels using qRT–PCR (Figure 1d). qRT–PCR showed a significantly higher expression of *bla*_B_ than *bla*_GOB_ in the WT strain. The deletion of one gene did not alter the expression of the other. In addition, the expressions of both *bla*_B_ and *bla*_GOB_ were unaffected by imipenem exposure.

The putative roles of *bla*_B_ and *bla*_GOB_ in increased carbapenem MICs were conflicting in the previous literature (Table S1); most studies showed that both *bla*_B_ and *bla*_GOB_ contributed equally to the increase of carbapenem MICs for *E. coli*. Our results of MIC testing, enzymatic activity assays and *in vivo* experiments evaluating *E. anophelis* showed a significant contribution of *bla*_B_ to high carbapenem MICs that may mask the modest contribution of *bla*_GOB_; this finding may be partly attributable to the higher expression level of *bla*_B_. Previous literature (Table S1) disclosed that ceftazidime susceptibility was reduced by all three β-lactamases and associated *bla*_CME_ with increased aztreonam MICs. However, our study showed that *bla*_CME_ was the only gene associated with high ceftazidime MICs and had no effect on aztreonam MICs (Table 1). The discrepancy between the results of our study and those of previous reports may be attributable to host factors (*E. coli* versus *E. anophelis*), the use of different subtypes of each β-lactamase gene^10^ and dissimilar expression levels of β-lactamase genes pertinent to vectors or promoters used in the earlier studies.

### Conclusions

The constitutional production of β-lactamase B in *E. anophelis* hydrolysed carbapenems and led to increased carbapenem MICs and a lack of *in vivo* efficacy of imipenem. The *bla*_B_ gene was also responsible for increased MICs of ampicillin/sulbactam and piperacillin/tazobactam. The *bla*_CME_ gene was associated with increased MICs of ceftazidime and cefepime.

## Supplementary Material

dkae137_Supplementary_Data

## Supplementary data

Table S1 is available as Supplementary data at *JAC* Online.
