# Supplementary material for: The individual contributions of blaB, blaGOB and blaCME on MICs of β-lactams in Elizabethkingia anophelis
Source: J Antimicrob Chemother. 2024 May 13;79(7):1577–80. doi: 10.1093/jac/dkae137 (PMC11215548; doi:10.1093/jac/dkae137)
Supplement: dkae137_Supplementary_Data [file dkae137_supplementary_data.docx]

**Supplementary Table S1. Contribution of individual β-lactamase genes to MIC (mg/L) change of β-lactams in the previous literature.**

| β-lactamase gene | Antibiotics tested | Bellais et al., 2000.^a, b^ | |  | Chang et al., 2021.^c^ | |  | Hu et al., 2020.^d^ | |
| --- | --- | --- | --- | --- | --- | --- | --- | --- | --- |
|  |  | MIC change | Fold change |  | MIC change | Fold change |  | MIC change | Fold change |
| *bla*_B_ | Imipenem | 0.12 to 0.5 | 4 |  | 0.125 to 4 | 32 |  | 0.03 to 32 | 1024 |
|  | Meropenem | 0.06 to 0.12 | NS^h^ |  |  |  |  | 0.12 to 16 | 128 |
|  | Ceftazidime | 0.5 to 0.5 | NS |  | 1 to 4 | 4 |  | ≤1 to >16 | ≥16 |
|  | Cefepime | 0.03 to 0.03 | NS |  | <0.125 to <0.125 | NS |  | ≤2 to ≤2 | NS |
|  | Aztreonam | 0.25 to 0.25 | NS |  | <0.125 to <0.125 | NS |  | ≤2 to ≤2 | NS |
|  |  |  |  |  |  |  |  |  |  |
| *bla*_GOB_ | Imipenem | 0.12 to 0.5 | 4 |  | 0.125 to 2 | 16 |  | 0.03 to 32 | 1024 |
|  | Meropenem | 0.06 to 0.12 | NS |  |  |  |  | 0.12 to 32 | 256 |
|  | Ceftazidime | 0.5 to 16 | 32 |  | 1 to 16 | 16 |  | ≤1 to >16 | ≥16 |
|  | Cefepime | 0.03 to 0.06 | NS |  | <0.125 to <0.125 | NS |  | ≤2 to ≤2 | NS |
|  | Aztreonam | 0.25 to 0.25 | NS |  | <0.125 to <0.125 | NS |  | ≤2 to ≤2 | NS |
|  |  |  |  |  |  |  |  |  |  |
| *bla*_CME_ | Imipenem | 0.12 to 0.25 | NS |  | 0.125 to 0.25 | NS |  |  |  |
|  | Meropenem |  |  |  |  |  |  |  |  |
|  | Ceftazidime | 0.5 to 32 | 64 |  | 1 to 64 | 64 |  |  |  |
|  | Cefepime | 0.03 to 0.5 | 16 |  | <0.125 to <0.125 | NS |  |  |  |
|  | Aztreonam | 0.25 to 16 | 64 |  | <0.125 to 8 | >64 |  |  |  |

| β-lactamase gene | Antibiotics tested | Hu et al., 2020.^e^ | |  | Colapietro et al., 2016.^f^ | |  | Yasmin et al., 2023^g^ | |
| --- | --- | --- | --- | --- | --- | --- | --- | --- | --- |
|  |  | MIC change | Fold change |  | MIC change | Fold change |  | MIC change | Fold change |
| *bla*_B_ | Imipenem | ≤0.25 to 0.5-8 | >2 ^i^ |  | 0.12 to 16 | 128 |  | 0.01 to >8 | >512 |
|  | Meropenem | ≤0.13 to 0.25-4 | >2 ^i^ |  | 0.12 to 16 | 128 |  |  |  |
|  | Ceftazidime | ≤1 to 4-16 | ≥4 |  | 012 to 2 | 16 |  | 1 to >64 | >64 |
|  | Cefepime | ≤1 to ≤1 | NS |  | 0.06 to 0.5 | 8 |  |  |  |
|  | Aztreonam | ≤2 to ≤2 | NS |  | 0.06 to 0.12 | NS |  | <2 to <2 | NS |
|  |  |  |  |  |  |  |  |  |  |
| *bla*_GOB_ | Imipenem | ≤0.25 to ≤0.25-8 | >2 ^i^ |  |  |  |  |  |  |
|  | Meropenem | ≤0.13 to ≤0.13-0.5 | >2 ^i^ |  |  |  |  |  |  |
|  | Ceftazidime | ≤1 to >32 | >32 |  |  |  |  |  |  |
|  | Cefepime | ≤1 to ≤1 | NS |  |  |  |  |  |  |
|  | Aztreonam | ≤2 to ≤2 | NS |  |  |  |  |  |  |

^a^ Bellais S, Poirel L, Naas T et al. Genetic-biochemical analysis and distribution of the Ambler class A beta-lactamase CME-2, responsible for extended-spectrum cephalosporin resistance in *Chryseobacterium* (*Flavobacterium*) *meningosepticum*. *Antimicrob Agents Chemother* 2000; **44:** 1-9.

^b^ Bellais S, Aubert D, Naas T et al. Molecular and biochemical heterogeneity of class B carbapenem-hydrolyzing beta-lactamases in *Chryseobacterium meningosepticum*. *Antimicrob Agents Chemother* 2000; **44:** 1878-86.

^c^ Chang Y, Zhang D, Niu S et al. MBLs, rather than efflux pumps, led to carbapenem resistance in fosfomycin and aztreonam/avibactam resistant *Elizabethkingia anophelis*. *Infect Drug Resist* 2021; **14:** 315-27.

^d^ Hu R, Zhang Q, Gu Z. Whole-genome analysis of the potentially zoonotic *Elizabethkingia miricola* FL160902 with two new chromosomal MBL gene variants. *J Antimicrob Chemother* 2020; **75:** 526-30.

^e^ Hu R, Zhang Q, Gu Z. Molecular diversity of chromosomal metallo-β-lactamase genes in *Elizabethkingia* genus. *Int J Antimicrob Agents* 2020; **56:** 105978.

^f^ Colapietro M, Endimiani A, Sabatini A et al. *Bla*_B-15_, a new *bla*_B_ metallo-β-lactamase variant found in an *Elizabethkingia miricola* clinical isolate. *Diagn Microbiol Infect Dis* 2016; **85:** 195-7.

^g^ Yasmin M, Rojas LJ, Marshall SH et al. Characterization of a novel pathogen in immunocompromised patients: *Elizabethkingia anophelis*-exploring the scope of resistance to contemporary antimicrobial agents and β-lactamase inhibitors. *Open Forum Infect Dis* 2023; **10:** ofad014.

^h^ NS, not significant, indicating an MIC change within 2 folds, or an MIC below detection limit after acquisition of the target gene.

^i^ The change varied in isolates with different subtypes of β-lactamase genes; some had no effect.
